# Overview of the Pathogenesis, Genetic, and Non-Invasive Clinical, Biochemical, and Scoring Methods in the Assessment of NAFLD

**DOI:** 10.3390/ijerph16193570

**Published:** 2019-09-24

**Authors:** Viera Kupčová, Michaela Fedelešová, Jozef Bulas, Petra Kozmonová, Ladislav Turecký

**Affiliations:** 1 3rd Department of Internal Medicine, Faculty of Medicine, Comenius University, University Hospital, 83305 Bratislava, Slovakia; 2 1st Department of Internal Medicine, Faculty of Medicine, Comenius University, University Hospital, 81369 Bratislava, Slovakia; 3 Institute of Chemistry, Biochemistry and Clinical Biochemistry, Faculty of Medicine Comenius University, 81372 Bratislava, Slovakia

**Keywords:** nonalcoholic fatty liver disease (NAFLD), steatosis, nonalcoholic steatohepatitis (NASH), fibrosis, biochemical diagnostic, genetic diagnostic, non-invasive scoring methods

## Abstract

Nonalcoholic fatty liver disease (NAFLD) is the most prevalent chronic liver disease worldwide. It represents a range of disorders, including simple steatosis, nonalcoholic steatohepatitis (NASH), and liver cirrhosis, and its prevalence continues to rise. In some cases, hepatocellular carcinoma (HCC) may develop. The develop;ment of non-invasive diagnostic and screening tools is needed, in order to reduce the frequency of liver biopsies. The most promising methods are those able to exclude advanced fibrosis and quantify steatosis. In this study, new perspective markers for inflammation, oxidative stress, apoptosis, and fibrogenesis; emerging scoring models for detecting hepatic steatosis and fibrosis; and new genetic, epigenetic, and multiomic studies are discussed. As isolated biochemical parameters are not specific or sensitive enough to predict the presence of NASH and fibrosis, there is a tendency to use various markers and combine them into mathematical algorithms. Several predictive models and scoring systems have been developed. Current data suggests that panels of markers (NAFLD fibrosis score, Fib-4 score, BARD score, and others) are useful diagnostic modalities to minimize the number of liver biopsies. The review unveils pathophysiological aspects related to new trends in current non-invasive biochemical, genetic, and scoring methods, and provides insight into their diagnostic accuracies and suitability in clinical practice.

## 1. Background

Nonalcoholic fatty liver disease (NAFLD) does not present as a single disease; rather, it is a spectrum of conditions, ranging from simple steatosis and nonalcoholic steatohepatitis (NASH) to liver cirrhosis, with its complications including hepatocellular carcinoma (HCC). NAFLD is a systemic condition, featuring metabolic, cardiovascular, and (hepatic/extrahepatic) cancer risks [1,2]. NAFLD is the most frequent cause of chronic liver injury in adults in developed countries [3,4]. About one-quarter of fatty liver cases develop NASH and over one-quarter of NASH patients develop severe fibrosis [1,5,6,7]. NAFLD is a precursor to type 2 diabetes (T2DM) and metabolic syndrome, and progressive liver disease develops in T2DM patients in whom the course of the disease is worsened by NAFLD [2,8,9,10]. NAFLD is highly prevalent in certain cohorts of individuals, who are potentially amenable to selective screening strategies and intensive follow-up schedules for early identification of liver-related and extrahepatic complications and for which earlier and more aggressive treatment schedules should be carried out, whenever possible [2]. Liver biopsy is an invasive diagnostic tool with little but significant hazard, and the decision of when to perform it remains to be controversial [11,12,13]. Consequently, it is necessary to search for less invasive methods for screening, distinguishing various NAFLD stages, and following their progression [14,15]. Non-invasive diagnosis is based on clinical and biochemical markers, scoring models, and algorithms of methods which have sufficient sensitivity, specificity, and reproducibility [16,17,18]. As non-invasive diagnostic methods should reduce the frequency of liver biopsies, they have to focus on two targets: Differentiation of simple steatosis from steatohepatitis and staging of fibrosis [19,20,21]. The detection of liver inflammation and fibrosis does not have only predictive value, but is important for determining the treatment threshold [22,23,24].

## 2. Pathophysiology of NAFLD

**Figure 1:**
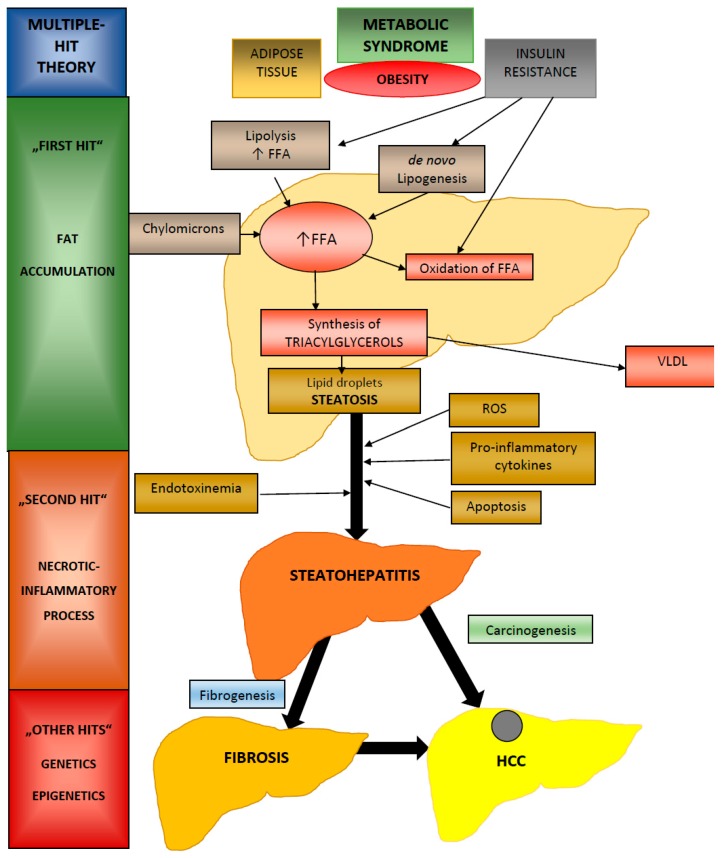
Pathogenesis of nonalcoholic fatty liver disease (NAFLD). Legend: FFA—free fatty acids, VLDL—very low density lipoproteins, ROS—reactive oxygen species, HCC—hepatocellular carcinoma.

Many diagnostic markers result from pathophysiological processes in hepatocytes which are typical of fatty liver injury, such as inflammation, cell death, and oxidative stress [25,26,27,28]. The pathogenesis of NAFLD is not completely known. The “multiple hit” hypothesis considers several insults acting together on genetically predisposed subjects to induce NAFLD and provides a more accurate explanation of NAFLD pathogenesis [29] (Figure 1). Such hits include insulin resistance, hormones secreted from adipose tissue, nutritional factors, gut microbiota, and genetic and epigenetic factors [29,30,31,32]. Obesity seems to have an important position in the development of NAFLD, but NAFLD occurs even in lean patients [33,34,35]. Obesity may lead to metabolic syndrome and insulin resistance (IR). On the other hand, insulin resistance may be responsible for NAFLD in non-obese patients [30,36,37,38].

The first hit leads to fat accumulation in hepatocytes as lipid droplets in the cytoplasm and causes simple steatosis. This state is reversible, and is associated with abnormal triglyceride storage. Triglycerides are produced from free fatty acids (FFAs). The main source of FFAs are plasmatic non-esterified fatty acids (NEFAs), followed by de novo lipogenesis and dietary fats, in the form of chylomicron lipoproteins. NEFAs especially arise from the lipolysis of the adipose tissue, which is induced by IR and during fasting [3,19]. The FFAs in the liver may follow three different pathways: Beta-oxidation (mainly in the mitochondria), the export of very low density lipoproteins (VLDL) into the blood with the help of apolipoprotein B (APOB), and synthesis of triglycerides [39]. It has been proposed that excessive intra-abdominal fat can induce excessive FFA reflux into the liver through the portal circulation. Triglyceride deposition in the form of lipid droplets (liver steatosis) may represent a vulnerable condition for the second hit, which causes hepatic inflammation and necrosis and can progress to fibrosis and cirrhosis. Excessive FFA oxidation can lead to oxidative stress with free radical formation and mitochondrial dysfunction, in general leading to the state known as lipotoxicity [40]. The immune response to lipotoxicity promotes inflammatory and wound-healing processes, which can lead to fibrogenesis and NAFLD progression [29]. On one hand, inflammatory pathways are activated in the liver, propelled by Kupffer´s cells (KCs), neutrophils, and natural killer cells (NK), as well as through the production of proinflammatory cytokines (e.g. interleukin 6 (IL-6), tumor necrosis factor alpha (TNF-α), and interleukin 1 (IL-1)). On the other hand, lipotoxicity also promotes inflammatory reaction in the adipose tissue and deregulates adipocytokine production, especially through the inhibition of adiponectin and the induction of leptin [41,42,43,44,45]. The second hit also includes apoptosis and gut-derived bacterial endotoxinemia, which play important roles in the development of NAFLD [40]. The microbiota can change the whole body’s lipid metabolism, as it can shift it from oxidation to de novo production [46,47,48,49,50]. NAFLD has been associated with small intestinal bacterial overgrowth and increased permeability, which can result in endotoxinemia and the activation of Kupffer cells [46]. The necroinflammatory stage, which is typical for NASH, leads to the activation of stellate hepatic cells, which are responsible for fibrogenesis, and progenitor cells, which promote hepatocarcinogenesis [51,52,53]. For instance, insulin resistance has been considered a promotor of stellate cell proliferation and an activator of collagen 1 production [36,54,55]. The third hit includes genetic factors. Different studies have shown familial aggregation and racial varieties in the prevalence of NAFLD and have mentioned a genetic predisposition to NAFLD [56]. The most evident genetic association is with palatine-like phospholipase 3 (PNPLA3), where certain non-synonymous single nucleotide polymorphisms (SNPs) have been associated with the severity of steatosis and the presence of NASH [57] (Table 1).

## 3. Clinical Evaluation of NAFLD 

The majority of subjects with NAFLD are clinically asymptomatic during the pre-cirrhotic stage. Patients can complain about fatigue and uncertain discomfort in the right upper abdominal quadrant. Physical examination can reveal hepatomegaly and obesity [58]. Secondary NAFLD involves complex pathophysiological and clinical consequences that ensue when the liver becomes an ectopic site of lipid storage, owing to reasons other than its mutual association with metabolic syndrome. Disorders affecting the gonadal hormones, thyroid hormones, or growth hormones (GH) may cause secondary forms of NAFLD to develop, which exhibit pathophysiologic features and, in theory, may affect the possibility of receiving effective treatment. Some common endocrine diseases, such as polycystic ovary syndrome (PCOS), hypothyroidism, hypogonadism, and GH deficiency, may be part of a naturally occurring disease model of NAFLD in humans [27,59]. As the disease progresses, features of liver decompensation can be present (e.g., jaundice, ascites, edema, gastrointestinal bleeding, and encephalopathy) [60]. Clinical features develop with the severity of the disease; thus, clinical symptoms are not crucial for making a diagnosis of early-stage NAFLD [61,62,63]. We should check for other signs related to metabolic syndrome, such as hypertension, diabetes mellitus, and abdominal obesity. to guide us in diagnosing NAFLD [2,64,65,66,67,68]. The diagnosis of NAFLD requires evidence of hepatic steatosis; in the absence of other causes of liver fat accumulation. NAFLD is often suspected in clinical practice when an individual with features of metabolic syndrome is found to have an increase in serum aminotransferase levels. Almost 80% of patients with NAFLD, however, have no biochemical abnormalities, which has several possible explanations [36,69]. The components of the metabolic syndrome are closely associated with NAFLD. Nearly two-thirds of people with obesity and T2DM and half of patients with hyperlipidaemia and hypertension have fat identified upon liver ultrasound. The presence of multiple features of the metabolic syndrome has been associated with more severe NAFLD-related liver disease and a higher likelihood of progression to NASH and liver fibrosis [24,70,71,72]. Ultrasonography is recommended as the first-line diagnostic method in assessing steatosis [15,17], while serum biomarkers and biomarker panels are alternative tools when imaging tools are not available in larger-scale screening studies [6,18,19,24]. An increasing number of biomarker panels have been used in clinical and research applications, while most have been validated in studies with relatively small populations, or in studies with sub-optimal gold criteria [15]. Therefore, future well-designed studies are needed to develop a more effective noninvasive biomarker panel for identifying NAFLD [6,17,20,21,23]. Magnetic resonance imaging-derived proton density fat fraction (MRI-PDFF) not only presents excellent performance for diagnosing NAFLD, but also accurately detects changes in fat content during disease progression [15]. However, MRI-PDFF is costly, time-consuming, and device dependent, which makes it difficult to achieve widespread application [15]. More effective, feasible, and easily operated tools are needed for diagnosing NAFLD, especially for early steatosis [15,17,24].

## 4. Laboratory Evaluation of NAFLD

### 4.1. Routine Markers of Liver Injury and Metabolic Syndrome

The majority of subjects with NAFLD are clinically asymptomatic during the pre-cirrhotic stage, but usually we can diagnose them through abnormal liver tests; mostly through increased levels of alanine-aminotransferase (ALT), aspartate aminotransferase (AST), and gamma-glutamyl transpeptidase (GGT). Hepatic enzymes are not reliable markers, as they do not have to be elevated even in advanced NAFLD [73,74,75]. Generally, the AST to ALT ratio increases with the severity of the necroinflammatory and fibrotic changes. In most cases, the serum prothrombin time, bilirubin level, and serum albumin level are normal, except in patients with NAFLD-associated cirrhosis. The serum ferritin level is elevated in more than 20% of NAFLD patients and can be a marker for advanced disease and increased mortality [15,76]. The iron serum is associated with oxygen radicals, which contributes to necroinflammation and fibrosis, two important parameters of NAFLD. Serum iron is higher in individuals with NASH than in those with simple steatosis. Serum ferritin exhibits a moderate performance in diagnosing NASH (AUROC 0.73). Through biopsies of NAFLD patients, a scoring system which combined serum ferritin with type IV collagen 7S and fasting insulin showed the ability to predict NASH with an AUROC of 0.78–0.85 [15]. High sensitivity C-reactive protein (CRP) is a generic inflammatory marker and is also correlated with NASH [77,78]. Impaired glucose metabolism (dysglycemia and impaired glucose tolerance), insulin resistance, hyperinsulinemia, dyslipidemia (hypertriglyceridemia), and hypercholesterolemia are associated with metabolic disorders related to NAFLD [17,79,80,81,82]. If NAFLD is suspected, laboratory findings can be used but they do not refer to histological severity; however, they can predict cardiovascular morbidity and mortality [83,84,85].

### 4.2. Markers of Inflammation

The correlation of NAFLD with general inflammatory markers such as CRP and ferritin has been described [86,87]. A novel acute phase reactant, plasma pentraxin 3 (PTX 3), seems to be promising for distinguishing variants of NAFLD. PTX 3 is increased in NASH, but also in other inflammatory states [88]. NAFLD is also associated with subclinical systemic inflammation and corresponds well with various cytokines and adipokines. A long-recognized proinflammatory cytokine is TNF-α, which is secreted by the adipose tissue of obese individuals, as well as hepatocytes, Kupffer cells, and other cells. TNF-α is highly expressed in NASH and, in general, enhances inflammation, necrosis, apoptosis, and fibrosis [15,43]. Despite this fact, it is one of the most studied cytokines, and numerous studies have confirmed its role in the pathogenesis of NAFLD. Anti-TNF therapy hampers the development of NAFLD. Other important cytokines to mention are interleukin (IL)-6, IL-1, IL-8, and IL-18. IL-6 seems to be the most related to NASH (Table 2). In a study by Tarantino et al., an area under the receiver-operating characteristics curve (AUROC) of 0.82 was shown [41,89]. Several studies have proved the importance of interleukins in pathological pathways of NAFLD, but their precise role is not still clear [32,41,43,89]. The relationship between their levels and stages of NAFLD is controversial and, as single markers, they are not useful enough [26,43]. The development of NAFLD and metabolic syndrome are associated with chemokines produced by adipose tissue, adipokines, which are also responsible for subclinical systemic inflammation in obese individuals [26,43,90]. Adiponectin seems to be a protective cytokine [90]. It is secreted only by the visceral adipose tissue of obese subjects and has anti-inflammatory, antiproliferative, antiatherogenic, and antihyperglycemic effects [42,90]. Adiponectin is a cytokine of key importance in NAFLD, which is able to regulate liver steatosis, insulin resistance, inflammation, and fibrosis [42,43,90,91]. Adiponectin levels are low in NAFLD patients, and they are a negative predictor of NASH in adults [91]. Other notable adipocytokines are leptin, ghrelin, visfatin, resistin, and retinol-binding protein 4 (RBP4). Leptin levels correlate with total body fat, and leptin resistance may be considerable in liver fibrogenesis [92]. Ghrelin is a peptide produced mainly by the stomach, which stimulates food intake. It is correlated with insulin sensitivity, and obese individuals have lower concentrations of de-acyl ghrelin [93]. Resistin and visfatin may play roles in obesity and insulin resistance, and higher plasma levels are found in obese subjects, but their importance in the pathogenesis of NAFLD is not still clear. Most cytokines associated with liver injury in NAFLD are not sensitive and specific enough for the staging of NAFLD; thus, more research in this field is needed [24].

### 4.3. Markers of Oxidative Stress

It is known that the increased production of reactive oxygen species (ROS) is responsible for lipid peroxidation, which leads to inflammation and fibrogenesis through stellate cell activation. The generation of ROS plays an important role in producing liver damage and initiating hepatic fibrogenesis [95,96]. Levels of thiobarbituric acid reactive substances (TBARS), nitric oxide (NO), superoxide dismutase (SOD), catalase, malonaldehyde (MDA), and vitamin E may be potential markers of disease, but it is not easy to determine their levels in serum as they are relatively volatile. Some studies have confirmed an association of markers of oxidative stress with NASH and liver fibrosis, especially TBARS, MDA, and oxidized VLDL, but results have been inconsistent [26].

### 4.4. Markers of Apoptosis

Cell apoptosis is related to defective permeabilization of the mitochondrial membrane and the release of proteins into the cytosol. A major intermediate filament protein in hepatocytes is cytokeratin 18 (CK 18). Several studies have proved the correlation of the CK 18 fragment level with the severity of NAFLD. Values of CK18 and its plasma fragments have shown an AUROC of 0.9 for detecting steatosis and 0.8 for detecting NASH [97,98]. CK 18 seems to be a reliable biomarker for hepatocellular injury; however, it has not yet been used in clinical practice. Another protein released during apoptosis is polypeptide-specific antigen, which could be a potential marker for fibrosis and could have clinical utility in the follow-up of obese patients with NASH [99].

### 4.5. Markers of Fibrogenesis

Fibrogenetic activity or extracellular matrix (ECM) turnover are typical changes in advanced-stage liver injury. Some of the most important factors in the process of extracellular matrix creation and fibrogenesis are changes in the activity of matrix metalloproteinases (MMP) and their specific inhibitors [100]. One of the most-studied matrix components HA, which is synthetized by stellate cells. Several studies have confirmed that serum hyaluronic acid is a reliable marker of hepatic fibrosis in NAFLD [94,101]. The enhanced liver fibrosis panel includes markers of fibrogenesis: Tissue inhibitor of metalloproteinase 1 (TIMP 1) and HA endaminoterminal peptide of procollagen III (P3NP). Other biomarkers for extracellular matrix turnover are transforming growth factor β, type IV collagen 7S domain, and endothelin-1 [102,103,104]. Proteomic analysis has also revealed other components of the extracellular matrix: Laminin and lumican. These markers have been proven to be predictors of fibrosis; however, they are not yet used in routine practice [89].

## 5. Differentiation of Steatosis, Steatohepatitis, and Fibrosis

As isolated non-invasive biochemical parameters are not specific and sensitive enough to predict the presence of NASH and fibrosis, there is a tendency to use various markers and to combine them into mathematical algorithms to avoid the use of invasive liver biopsy. Several predictive models have been developed and validated (Table 3). Non-invasive methods rely on two different approaches: A “biological” approach, based on the quantification of biomarkers in serum samples, or a “physical” approach, based on the measurement of liver stiffness, using either ultrasound or magnetic resonance-based elastography techniques. Although these approaches are complementary, they are based on different rationales. Liver stiffness corresponds to a genuine and intrinsic physical property of liver parenchyma, whereas serum biomarkers indicate several—not strictly liver-specific—clinical and serum parameters which are associated with NASH or the fibrosis state, as assessed by liver biopsy [15,17,105,106,107]. A widely used algorithm for the diagnosis of simple steatosis is the fatty liver index (FLI), which includes the body mass index (BMI), waist circumference (WC), and serum values of GGT and triglycerides. It has an AUROC of 0.834 for NAFLD [108,109,110]. Borman et al. described its limited use in obese patients with an AUROC of 0.67 [111]. A less-complicated model is the lipid accumulation product (LAP), which incorporates gender, WC, and triglyceridemia and has an AUROC for detecting steatosis of 0.79 [112]. The Hepatic Steatosis Index is another panel that includes gender, T2DM history, BMI, ALT, and AST and has an AUROC of 0.81 [113]. A novel model for predicting fatty liver disease is the ZJU index, which was validated in a Chinese population and includes BMI, fasting plasma glucose, triglycerides, and the ALT/AST ratio, which has an AUROC of 0.82 for diagnosing NAFLD and 0.896 for detecting steatosis [114]. The SteatoTest is based on the levels of α-2 macroglobulin, apolipoprotein A1, haptoglobin, total bilirubin, GGT, ALT, fasting glucose, triglycerides, cholesterol, age, gender, and BMI. This algorithm showed the worst AUROC, 0.71. In addition, it is not a cost-effective tool [115]. The AST to ALT ratio (AAR) is the simplest test for evaluating NAFLD and predicting fibrosis. In general, AAR ≥ 1 suggests the presence of advanced fibrosis or cirrhosis. McPherson et al. demonstrated that AAR can reliably exclude the presence of advanced fibrosis in patients with NAFLD using a cut-off value of 0.8, where AAR showed an AUROC of 0.83 [116]. The AST to platelet ratio index (APRI) is another elementary test which includes the AST level and platelet count and was originally used routinely for detecting chronic hepatitis C. Kruger et al. showed that APRI is an accurate bedside marker for advanced fibrosis, which can avoid the need for liver biopsy in patients with NAFLD; it showed an AUROC of 0.85 [117]. However, significantly lower AUROC values of 0.66–0.73 were confirmed in other studies [118,119]. The initials of the BARD score indicate that it incorporates BMI, AAR, and the presence of T2DM. The BARD score was validated on a cohort of Caucasian patients and was shown to have an AUROC of 0.82 and a high negative predictive value (NPV) of 97% [120]. Similar studies showed lower levels of accuracy, with AUROC values of 0.65–0.77 [116,119,121]. The NAFLD fibrosis score (NFS) is composed of six variables (age, hyperglycemia, BMI, platelet count, albumin, and AAR) and was developed in a large multicenter study of 733 patients, who were divided into two groups to estimate and validate the scoring panel. The presence of advanced fibrosis was diagnosed with high accuracy (positive predictive values of 90% and 82% in the estimation and validation groups, respectively), and liver biopsy was avoided in 75% of patients [122]. In a validation study on a Chinese population, the NPV was 91% and 79% of patients avoided liver biopsy. The highest value of AUROC (0.96) for the NAFLD fibrosis score was obtained by Demir et al. in a cohort of 267 patients [123]. The Nippon score (N score) gives the total number of risk factors and includes age, gender, history of T2DM, and hypertension. It showed an AUROC of 0.78 in a cohort of 182 Japanese patients with biopsy-confirmed NAFLD [124]. The BAAT score, which includes BMI, age, and ALT and triglyceride levels, gave an AUROC of 0.75 for moderate fibrosis and 0.92 for severe fibrosis in NAFLD patients [125]. The fibrosis-4 (FIB-4) test includes age, platelet count, and levels of ALT and AST, and was developed as a non-invasive panel to stage liver disease in subjects with HIV–hepatitis C virus (HCV) co-infection [126,127]. Several comparative studies showed that FIB-4 is the most promising diagnostic panel for distinguishing NASH from steatosis. The AUROC value for the FIB-4 index (0.87) was found to be superior to other scoring systems (NFS, APRI, AP index, AAR, BARD score, Nippon score: 0.86, 0.82, 0.81, 0.79, 0.76, and 0.71, respectively) for differentiating between advanced and mild fibrosis in 576 Japanese biopsy-proven NAFLD patients. Using the FIB-4 index, 58% of liver biopsies could be avoided [128]. The greatest AUROC value for FIB 4 (0.96), in comparison to the other panels, was obtained in a study of 165 Caucasian NAFLD patients [98]. Another study demonstrated that the FIB-4 test is the best predictive algorithm for advanced fibrosis, but it showed a smaller AUROC value, 0.80 [118]. The inferior diagnostic value of FIB 4 between noninvasive assessment systems was documented in a cohort of 228 Latin patients, with an AUROC value of 0.74 [119]. Demir et al. developed and validated the non-invasive Koeln–Essen index (NIKEI), which includes age, AAR, AST, and total bilirubin levels and showed an AUROC value of 0.97, compared with a value of 0.93 for FIB-4. The absence of severe fibrosis could be confirmed with a high level of accuracy (99–100%) [123]. The HAIR algorithm incorporates hypertension, ALT levels, and insulin resistance, and has been shown to be an independent marker for the diagnosis of NAFLD [129]. Using the HAIR model, Dixon et al. demonstrated 80% sensitivity and 89% specificity for NASH in patients after laparoscopic obesity surgery [130]. The fibrometer combines age, weight, fasting glucose, AST, ALT, ferritin levels, and platelet count. It is a test that was developed for staging fibrosis in chronic HCV (similar to other models), but has also been demonstrated to have excellent accuracy for the identification of NAFLD fibrosis, with an AUROC value of 0.94 [131]. The fibrotest (FT) is a patented formula that includes five serological markers: Haptoglobin, apolipoprotein A1, α2-macroglobulin, total bilirubin, and GGT levels. It was validated by Poynard et al. in a cohort of 761 patients with NAFLD and in morbidly obese patients with NAFLD, where it showed an AUROC value of 0.85 [115,132]. The NASH test includes gender, age, weight, height, and serum values of triglycerides, total cholesterol, ALT, AST, GGT, total bilirubin, α2-macroglobulin, haptoglobin, and apolipoprotein A1, and was shown to have an AUROC value of 0.79 for detecting NASH [133].

The following tests are also known as a super combination FibroMAX (BioPredictive, Paris, France) and are used in patients at risk of chronic liver diseases: FibroTest (BioPredictive) for the quantitative assessment of fibrosis; SteatoTest (BioPredictive) for the quantitative assessment of steatosis; ActiTest (BioPredictive) for the quantitative assessment of necroinflammatory activity in chronic viral hepatitis C and B; NashTest (BioPredictive) for the categorical diagnosis of nonalcoholic steatohepatitis; and AshTest for the quantitative assessment of alcoholic steatohepatitis (also known in the USA as HCV-FibroSURE, HBV-FibroSURE, ASH-FibroSURE, and NASH-FibroSURE; LabCorp, NC, USA) [135]. Palekar et al. constructed a composite index for distinguishing steatosis from NASH by summing the risk factors of age, gender, AST level, BMI, AAR, and HA. Its accuracy was shown with an AUROC value of 0.76, obtained in a small study of 80 patients [136]. The Antwerp NAFLD significant fibrosis takes in account WC, AST, and fasting C-peptide levels, as well as ultrasound steatosis scores, and was developed by a Belgian hepatologic group. It showed an AUROC value of 0.84 in a design cohort and 0.85 in a validation cohort [137]. The enhanced liver fibrosis panel (ELF) includes tissue inhibitor of matrix metalloproteinase 1 (TIMP1), HA, and the aminoterminal peptide of procollagen III. Guha et al. validated the Original European Liver Fibrosis panel (OELF) and a simplified algorithm not containing age, the Enhanced Liver fibrosis panel (ELF), in an independent cohort of 196 patients with NAFLD. The ELF panel showed an AUROC of 0.90 for distinguishing severe fibrosis, 0.82 for moderate fibrosis, and 0.76 for no fibrosis [134]. The NAFIC score is another predictive model, designed for a Japanese population, which combines serum values of ferritin, insulin, and type IV collagen 7S, which had an AUROC of 0.79 [138].

## 6. Genetical Evaluation and Multi-Omics Profiles of NAFLD

NAFLD development is influenced by environmental factors, such as dietary habits and sedentary lifestyle, but requires background knowledge of genetic susceptibility [139,140,141]. Heritability also explains inter-individual and racial differences of NAFLD occurrence and progression to NASH. Indeed, a recent large study showed that the heredity level of NAFLD is 26–27% [56,142]. Genetic studies include genome-wide association studies (GWAS) and candidate gene studies. GWAS are hypothesis-free studies that identify the genetic influences on common diseases, testing associations of common variants in the human genome and polymorphic traits [139]. Candidate gene studies originate from genomic, proteomic, or other studies, and just a single nucleotide polymorphism (SNP) is selected to investigate the role of a candidate gene in a pathogenetic mechanism.

The most relevant genetic association is with palatine-like phospholipase domain, containing 3 (PNPLA3); also known as adiponutrin [57,142]. Romeo et al. demonstrated a correlation of the PNPLA3 I148M variant, non-synonymous SNP-rs 738409, with increased liver fat content [143]. However, although there was a strong link between the PNPLA3 I148M variant and NAFLD, some meta-analyses did not confirm the association of this variant with metabolic syndrome and its features [144]. Studies have also documented an interplay between PNPLA3 variant I148M and advanced fibrosis in patients with NASH, the risk of HCC development, and the presence of chronic kidney disease [145,146,147]. The frequency of the 148M allele has also been shown to vary with ethnicity [143]. Another gene found by GWAS to be a risk factor of NAFLD is the transmembrane 6 superfamily member 2 (TM6SF2), nonsynonymous variant rs58542926. Kozlitina et al. identified the association between this SNP and the hepatic triglyceride content, elevated aminotransferases, and reduced serum levels of triglycerides and LDL-cholesterol [148]. Dongiovanni et al. showed that carriers of this variant had lower values of serum lipids and more severe steatosis and fibrosis than non-carriers. In fact, they seemed to be at risk of NASH progression [149]. Other genetic variants revealed by GWAS are neurocan (NCAN-rs2228603), protein phosphatase 1, regulatory (inhibitor) subunit 3B (PPP1R3B-rs4240624), glucokinase regulator (GCKR-rs780094), and lysophospholipase-like 1 (LYPLAL1-rs12137855) [56]. Gene candidate studies have suggested a vast number of SNPs as potential genetic factors that influence the development of NAFLD and the progression of NASH [139]. However, multiple studies have found that the factor most correlated with NAFLD is the PNPLA3 gene variant I148M. The identification of these variants does not have routine utility, but has elucidated some pathogenetic mechanisms of NAFLD [32].

Epigenetic mechanisms and their influences on gene expression in the pathogenesis of NAFLD have been discussed [44,150]. Epigenetic mechanisms are heritable, reversible, and modulated by environmental stimuli; they include histone acetylation and deacetylation, DNA methylation, microRNAs, and chromatin remodeling [151,152]. MicroRNAs (miRNAs) are the most important, which regulate gene expression and protein translation. They can also be called oncogenes or tumor suppressors, because of their roles in carcinogenesis [153,154]. The most commonly expressed miRNA in the human liver is miR-122, which is involved in NAFLD and is associated with a high risk of developing HCC [154]. Epigenetics has presented potential diagnostic, prognostic, and therapeutic targets [155,156].

Multiomics profiles include genomics, proteomics, metabolomics, lipidomics, and glycomics, and these have common characteristics: They involve hypothesis-free, large-scale analyses of specific serum or tissue products during a disorder and are used, in particular, as novel biomarkers. The first proteomic profile of NAFLD was performed on a cohort of 98 obese patients, and from 12 significant protein peaks, just fibrinogen γ showed a relation to fibrosis [157]. A later study discovered 15 proteins connected to NASH and advanced fibrosis, and two diagnostic models were developed for staging NAFLD. The first model was composed of six proteins (fibrinogen β chain, retinol binding protein 4, serum amyloid P component, lumican, transgelin 2, and CD5 antigen-like), and the second involved three proteins (component C7, insulin-like growth factor acid labile subunit, and transgelin 2); the AUROC values ranged from 0.83–0.91 for different stages of NAFLD [158]. Due to multiomic studies, numerous biomarkers have been revealed to be associated with NAFLD, as they are involved in necroinflammation and fibrogenesis [159,160,161,162,163,164,165].

## 7. Conclusions

Numerous non-invasive methods have been developed to cover the whole spectrum of NAFLD disorders, but only some of them are reliable for differentiating among steatosis, steatohepatitis, and the degree of fibrosis and for quantifying steatosis and, thus, fulfilling the criterion of minimizing the need for liver biopsy. The most promising, widely available, and easily applicable strategies to exclude advanced liver fibrosis seem to be scoring systems (e.g., FIB-4 score, NAFLD fibrosis score, and BARD score).

As the prevalence of NAFLD has risen worldwide, and as NAFLD has been associated with increased mortality owing to cardiovascular diseases followed by cancer and liver-related causes, it is important to identify patients at high risk of NAFLD [166,167,168]. There has recently been extensive development of noninvasive methods in the NAFLD field, from serum biomarkers and imaging to omics. [169,170]. Fatty liver is a common feature of different types of liver disease. The sensitivity and specificity of ultrasonography for diagnosing fatty liver are variable. A semiquantitative ultrasound score—the ultrasonographic fatty liver indicator (US-FLI)—has been closely associated with metabolic/histological variables in patients with NAFLD. In a study by Ballestri and al. (2017), US-FLI was correlated with the steatosis percentage in each liver disease group, as well as with lobular inflammation, ballooning, portal fibrosis, grading, and staging in patients with NAFLD or HCV. US-FLI was also correlated with waist circumference, body mass index, and insulin resistance. US-FLI accurately identified the histological severity and was correlated with metabolic parameters in patients with various steatogenic liver diseases [171]. Our ability to identify NAFLD patients and to estimate steatofibrosis with various ultrasound-based techniques has undergone tremendous progress over the last few years. However, it is more difficult to capture the inflammatory component of NASH with such ultrasound-assisted techniques. Moreover, semi-quantitative, quantitative, elastographic, and contrast-enhanced ultrasound techniques are increasingly being appreciated and made available; but not all such techniques will gain success in the clinical and research areas. Therefore, further research will precisely define the roles of the most innovative ultrasonographic techniques, while reducing costs and increasing feasibility [172,173]. The diagnostic accuracy of transient hepatic elastography [174,175,176], real-time elastography [177,178], shear-wave elastography, acoustic radiation force impulse imaging (ARFI) [179,180], MR elastography [181,182,183], 1H magnetic resonance spectroscopy [184], magnetic resonance imaging-proton density fat fraction, and other imaging methods in nonalcoholic fatty liver diseases have been evaluated [169,179,185,186,187] (Table 4).

New sequential combinations of non-invasive fibrosis tests and imaging methods may provide an accurate diagnosis of advanced fibrosis [112,187,188,189,190,191,192,193,194]. Further testing and validation are needed for non-invasive diagnosis and its use in clinical practice.

## Figures and Tables

**Table 1:** Summary of single nucleotide polymorphisms (SNPs) related to nonalcoholic fatty liver disease (NAFLD).

**Genes incorporated in Glucose and Lipid Metabolism**	
Apolipoprotein C III	*APOC3 rs 2854116, rs 2854117*
Peroxisome proliferative activated receptor α, γ, peroxisome proliferator-activated receptor γ coactivator 1-α	*PPAR α, rs 1800206, PPAR γ, rs1801282, PPARGC1A, rs2290602*
Fatty acid transport protein	*FATP5, rs 56225452*
Adiponectin	*ADIPQ, rs2241766, rs 1501299*
Leptin receptor	*LEPR rs62589000, rs6700986*
Resistin	*RETN rs 3745367*
**Genes incorporated in the pathogenesis of NAFLD**	
TNF-α, TNF-α related apoptosis inducing ligand	*TNF-α rs 1800629, rs361525,TRAIL rs6763816, rs4491934*
Toll like receptor	*TLR4 rs4986790*
Superoxide dismutase 2	*SOD2 rs4880*
Cytochrome P450 2E1	*CYP2E1 rs2031920*
Kruppel-like factor 6	*KLP6 rs3750816*
Transforming growth factor β1	*TGF-β1 rs1800471*
Angiotensin II, angiotensin II Type receptor	*AGII rs699, AGTR1 rs3772622, rs 3772633*

**Table 2:** Values of AUROC for the most accurate non-invasive biochemical methods.

Method	Field of Detection	Accuracy	Strengths	Advantages and Limitations	Reference
Biochemical Methods					
**IL-6**	NASH fibrosis	**AUROC** 0.83	95% CI: 0.67;0.98 *p* = 0.0024 Sensitivity 85% Specificity 86%.	Discrimination between advanced fibrosis patients compared to mild fibrosis patients and no fibrosis patients; *p* < 0.001.	[89]
**VCAM-1**	NASH fibrosis	**AUROC** 0.87 0.79 0.53	95% CI: 0.75;1.0 *p* = 0.0005 95% CI: 0.63;0.95 *p* = 0.0064 95% CI: 0.35;0.71 n.s.	Distinguish between advanced fibrosis and no fibrosis. Distinguish between mild fibrosis from advanced fibrosis. Poor sensitivity for distinguish in no fibrosis compared to mild fibrosis. In children and adolescents is elevated with obesity.	[78]
**HA**	NASH fibrosis	**AUROC** 0.94	Cut off 25 ug/L sensitivity 90%, specificity 84% CI: 0.59–0.99.	Discrimination between significant liver fibrosis F3 + F4 and mild to moderate, or no fibrosis (F0–F2); *p* < 0.001.	[94]
**Cytokeratin 18**	NASH fibrosis	M65 **AUROC** 0.89	Cut off 750 U/L, sensitivity 80%, specificity 82%, 95% CI: 0.57–0.95. Cut-off 211 U/L, sensitivity 0.79, Specificity 0.76, 95% CI: 0.56–0.93.	Diferentiation of patients with and without NASH. M65 *p* < 0.014, M30 *p* < 0.001. Can predict the disease severity in NASH patients.	[94]
		M30 **AUROC** 0.85	Cut off 750 U/L, sensitivity 80%, specificity 82%, 95% CI: 0.57–0.95. Cut-off 211 U/L, sensitivity 0.79, Specificity 0.76, 95% CI: 0.56–0.93.	Diferentiation of patients with and without NASH. M65 *p* < 0.014, M30 *p* < 0.001. Can predict the disease severity in NASH patients.	[15]

Legend: IL-6—interleukin 6, VCAM-1—vascular cell adhesion protein 1, HA—hyaluronic acid, CI—confidence interval, AUROC—area under the receiver-operating characteristic curve.

**Table 3:** Values of AUROC for most accurate non-invasive scoring methods.

Method	Field of Detection	Parameters Used for Calculation	Accuracy	Strengths	Advantages and Limitations	Ref.
Scoring Method						
**Fatty liver index** **(FLI)**	NAFLD	BMI, WC, GGT, triglycerides	**AUROC** **0.83** **AUROC** **0.67**	Optimal cut-off point 30 Sensitivity 79.8% Specificity 71.5% 95 % CI:0.825–0.842, *p* < 0.001.	Low cutoff of 30 is used to rule out NAFLD (negative likelihood ratio 0.2). High cutoff of 60 is used (with a positive likelihood ratio of 4.3). Poorly distinguishes moderate-to severe steatosis from mild steatosis. Limited use in obese patients.	[108] [111]
**Hepatic steatosis index** **(HSI)**	NAFLD	Gender, Diabetes mellitus, BMI, ALT/AST ratio	**AUROC** 0.81	Cut-off point 30 *p* < 0.001 Sensitivity of 93.1% Specificity of 92.4% (95 % CI: 0.81–0.824).	At values of <30, ruled out NAFLD. At values of >36, detected NAFLD. Poorly distinguishes moderate-to severe steatosis from mild steatosis. HSI accuracy decreases in obese children.	[113] [15]
**SteatoTest**	Steatosis	apha-2-macroglobulin, apolipoprotein A1, haptoglobin, bilirubin, GGT, ALT, glucose, triglycerides, cholesterol, age, gender, BMI	**AUROC** 0.71	At the cut off 0.38: Sensitivity 89.7% Specificity 44.9% PPV 90.9% NPV 41.3% PPV 92.4% for the dg. of steatosis >S0 using 0.38 cut off. NPV 59.3% for the dg. of steatosis >S1 using 0.69 cut off.		[115]
**NAFL Screening score**	NAFLD	Age, glucose, BMI, triglycerides, ALT/AST, uric acid	**AUROC** 0.87	At the cut-off 0.24: Sensitivity 92%; NPV 95% At the cut-off 0.44: Specificity 90%; PPV 84%		[15]
**NAFLD fibrosis score** **(NFS)**	Advanced fibrosis	Age, BMI, impaired fasting glucose and/or diabetes, AST/ALT ratio, platelet count, and albumin	**AUROC** 0.96 0.83 for cirrhosis 0.73 for advanced fibrosis 0.72 for significant fibrosis	At the cutoff ≤−1.455: Sensitivity 75% Specificity 93% PPV 63%; NPV 96%	Below the lower cutoff (≤−1.455), healthy. Above the cutoff (≥0.676), advanced fibrosis.	[123]
				At the cut- off ≥0.676: Sensitivity 19% Specificity 100% PPV100%; NPV 89%	Can be used to identify those at low or high risk for advanced fibrosis or cirrhosis.	[15]
**APRI**	Advanced fibrosis	AST/platelet ratio index	**AUROC** 0.85	Optimal cut off 0.98 Sensitivity of 75% Specificity of 86% PPV 54%; NPV 93%		[117]
			**AUROC** 0.75 for advanced fibrosis or cirrhosis 0.70 for significant fibrosis		Low specificity to diagnose advanced fibrosis.	[15]
**FIB-4**	Advanced fibrosis	Age, platelet count, ALT, AST	**AUROC** 0.85 for cirrhosis 0.80 for advanced fibrosis 0.75 for significant fibrosis	At the cut-off 1.3 Sensitivity 85% Specificity 65% PPV 36%; NPV 95% At the cut off 3.25 Sensitivity 26% Specificity 98% PPV 75%; NPV 85%	Can be used to identify patients at low or high risk for advanced fibrosis or cirrhosis.	[15]
**BARD score**	Advanced fibrosis	AST, ALT, BMI and diabetes	**AUROC** 0.70 for cirrhosis 0.73 for advanced fibrosis 0.64 for significant fibrosis		Low specificity to diagnose significant fibrosis and cirrhosis.	[15]
**Enhanced liver fibrosis** **(ELF)**	Advanced fibrosis Mild fibrosis Fibrosis not present	TIMP1, HA, aminoterminal peptide of pro-colagen III	**AUROC** 0.90 for severe fibrosis 0.82 for moderate fibrosis 0.76 for no fibrosis			[134]
**Hepatic steatosis index** **(HIS)**	Steatosis	Gender, T2DM, BMI, ALT, AST	**AUROC** 0.81	Sensitivity of 93.1%, at values of <30 ruled out NAFLD. Specificity of 92.4%, at values of >36 detected NAFLD.		[113]

Legend:, CI—confidence interval, GGT—gamma-glutamyl transpeptidase, BMI—body mass index, WC—waist circumference, TIMP1—tissue inhibitor of matrix metalloproteinase 1, T2DM—type 2 diabetes mellitus, APRI—AST to platelet ratio index, AUROC—area under the receiver-operating characteristic curve, NPV—negative predictive value, PPV—positive predictive value.

**Table 4:** Values of area under the receiver-operating characteristics curve (AUROC) for the most accurate imaging methods.

Method	Field of Detection	AUROC	Ref.
Imaging Methods			
USG	Steatosis	0.93	[169]
CT	Steatosis	0.92	[186]
MRI	Steatosis	0.99	[186]
TE	Advanced fibrosis	0.99	[179]
ARFI	Advanced fibrosis	0.97	[179]

Legend: USG-ultrasonography, CT-computer tomography, MRI-magnetic resonance imaging, TE-transient elastography, ARFI-acoustic radiation force impulse.
